# Supplementary material for: 3D Printing of Hierarchical Structures Made of Inorganic Silicon-Rich Glass Featuring Self-Forming Nanogratings
Source: ACS Nano. 2024 Oct 9;18(43):29748–59. doi: 10.1021/acsnano.4c09339 (PMC11526370; doi:10.1021/acsnano.4c09339)
Supplement: Supplementary file 1 — nn4c09339_si_001.pdf [file nn4c09339_si_001.pdf]

Supporting Information

3D printing of hierarchical structures made of inorganic silicon-rich glass featuring self-forming nanogratings

Po-Han Huang[1], Shiqian Chen[2], Oliver Hartwig[3], David E. Marschner[1], Georg S. Duesberg[3], Göran Stemme[1], Jiantong Li[2], Kristinn B. Gylfason[1], and Frank Niklaus[1,]*

[1] Division of Micro and Nanosystems, School of Electrical Engineering and Computer Science, KTH Royal Institute of Technology, Stockholm 10044, Sweden

[2] Division of Electronics and Embedded Systems, School of Electrical Engineering and Computer Science, KTH Royal Institute of Technology, Kista 16440, Sweden

[3] Institute of Physics, EIT 2, Faculty of Electrical Engineering and Information Technology, University of the Bundeswehr Munich & SENS Research Center, Neubiberg 85577, Germany

[*] Corresponding author (Email: frank@kth.se)

The Supporting Information includes:

Supporting Section 1

Supporting Figures 1-8

Supporting Table 1

References

Supporting Section 1. Material characterization

To characterize the elementary composition and chemical bonds of the 3D-printed glass, we collected its energy-dispersive X-ray (EDS), Raman, and photoluminescence (PL) spectra. The EDS spectrum of the printed glass showed the peaks of silicon (Si), oxygen (O), and carbon (C), while EDS is incapable of detecting hydrogen by principle (**Figure 3a, b** and **Figure S3**). The reported values of the atomic ratios of the elements in the material are only useful for qualitative analysis as discussed in METHODS. Compared to the EDS spectrum of the pristine HSQ, the O/Si ratio and the C/Si ratio of the printed glass were both lower. The carbon content observed in the printed glass was at the same level as that observed in the fused silica substrate, and thus is considered as a common residual contamination originating from the environment. The Raman spectrum of the printed glass showed a dominant peak at ~496 cm^{-1} which can originate from 4-membered Si-O rings in the silica glass network[1] and Si-Si bonds[2] (**Figure 3c**). We also observed characteristic Raman features of fused silica glass such as the broad peak between 120 and 580 cm^{-1}, the 3-membered Si-O rings at ~605 cm^{-1}, and the Si-O-Si bending at ~805 cm^{-1} in the spectrum of the printed glass.[3] Finally, we did not observe any feature around 2260 cm^{-1} which is the spectral position of the signature Si-H peak of pristine cage-form HSQ (**Figure S4**). This indicates the occurrence of transformation of the pristine cage-form HSQ to the crosslinked network form upon femtosecond-laser exposure. In the PL spectrum of the printed glass, a broad and asymmetric peak between 1.3 and 2.6 eV was observed (**Figure 3d**). The broad peak can be fitted by three peaks at ~1.6 eV from Si nanoclusters, at ~1.9 eV from non-bridging oxygen hole centers (NBOHC), and at ~2.55 eV possibly from hydrogen-related Si species.[4–7] EDS, Raman, and PL spectra of the glass structures printed with the laser polarization in parallel (Pol-X) and perpendicular (Pol-Y) to the writing direction showed no differences.

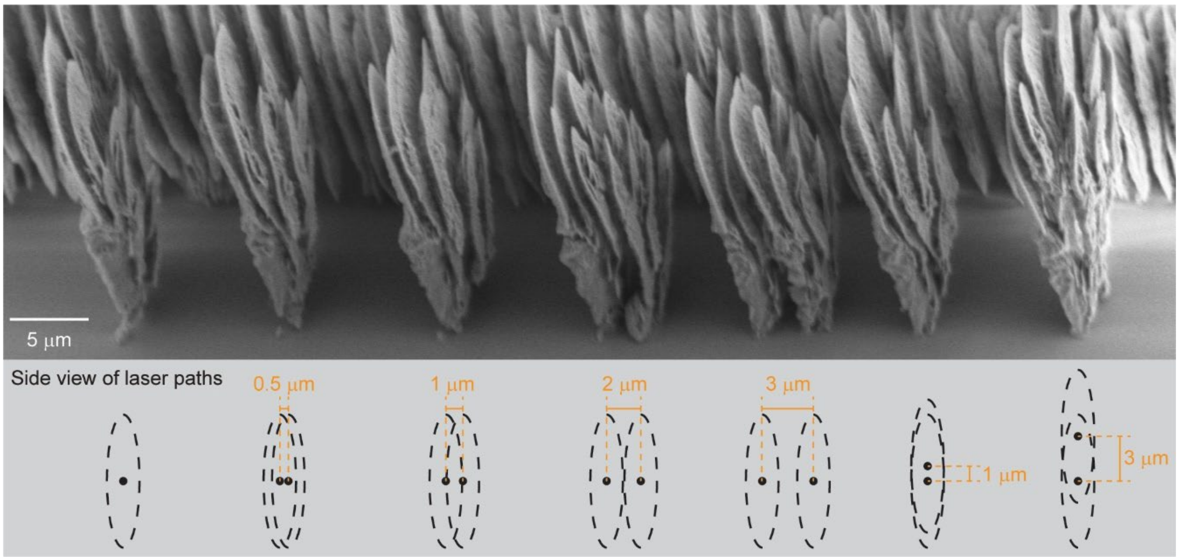

Supporting Figure 1. Investigation of the contact regions between nanoplates in self-forming nanogratings. Tilted-view scanning electron microscopy (SEM) image of the side wall of seven independent 3D-printed line structures with schematics below showing the side view of their corresponding laser writing paths. For the structure to the left a single line laser writing path was used, while for each of the remaining structures two lines for the laser writing path were used. In the schematics, one line is represented by one dashed ellipse with one dot marking the center of the ellipse, and the distance between two lines in each two-line structure is annotated. We observed that the nanoplates in one nanograting (i.e., the nanograting formed within one laser written line) were connected at the bottom. In addition, when the distance between two lines of the laser writing path is larger than 2 μm, the contact region of the two self-forming nanogratings started to separate. For instance, two separate "tips" appeared in the structure comprising two lines laterally separated by 3 μm, and an elongated contact region appeared in the structure comprising two lines vertically separated by 3 μm. Further investigation of the evolution of the contact regions in nanogratings with increasing number of overlapping laser writing paths is required to fully clarify the sizes and positions of contact regions in large 3D-printed hierarchical structures featuring self-forming nanogratings.

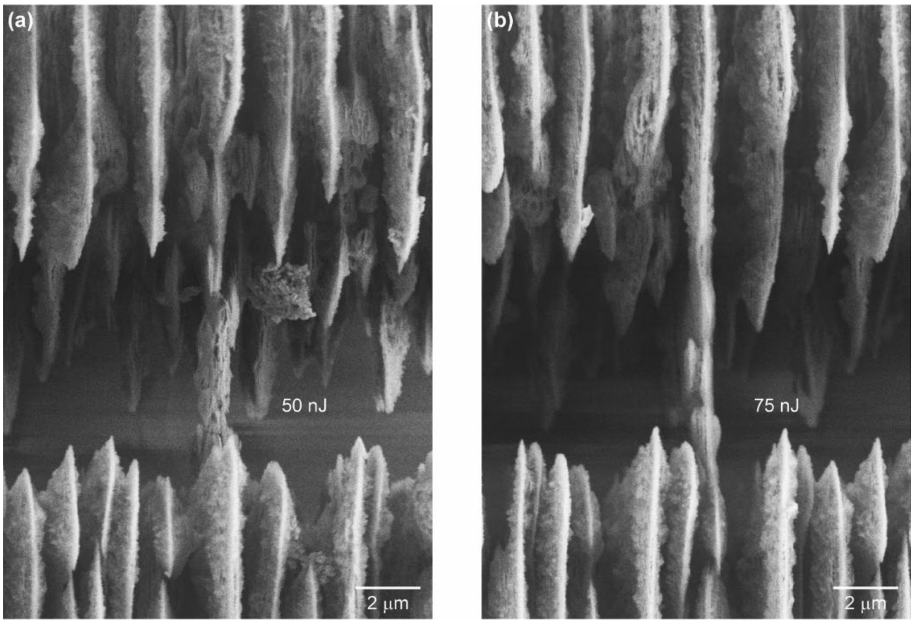

Supporting Figure 2. Single-line structures with minimum width. (a, b) Enlarged SEM images of the 3D-printed suspended single line structures that were printed using laser pulse energies of 50 and 75 nJ, respectively, shown in **Figure 1g**. Both structures were approximately 800 nm thick, while the structure in (a) was more porous than the structure in (b), which indicates that the laser dose was not sufficient for the complete formation of the nanogratings in the former case.

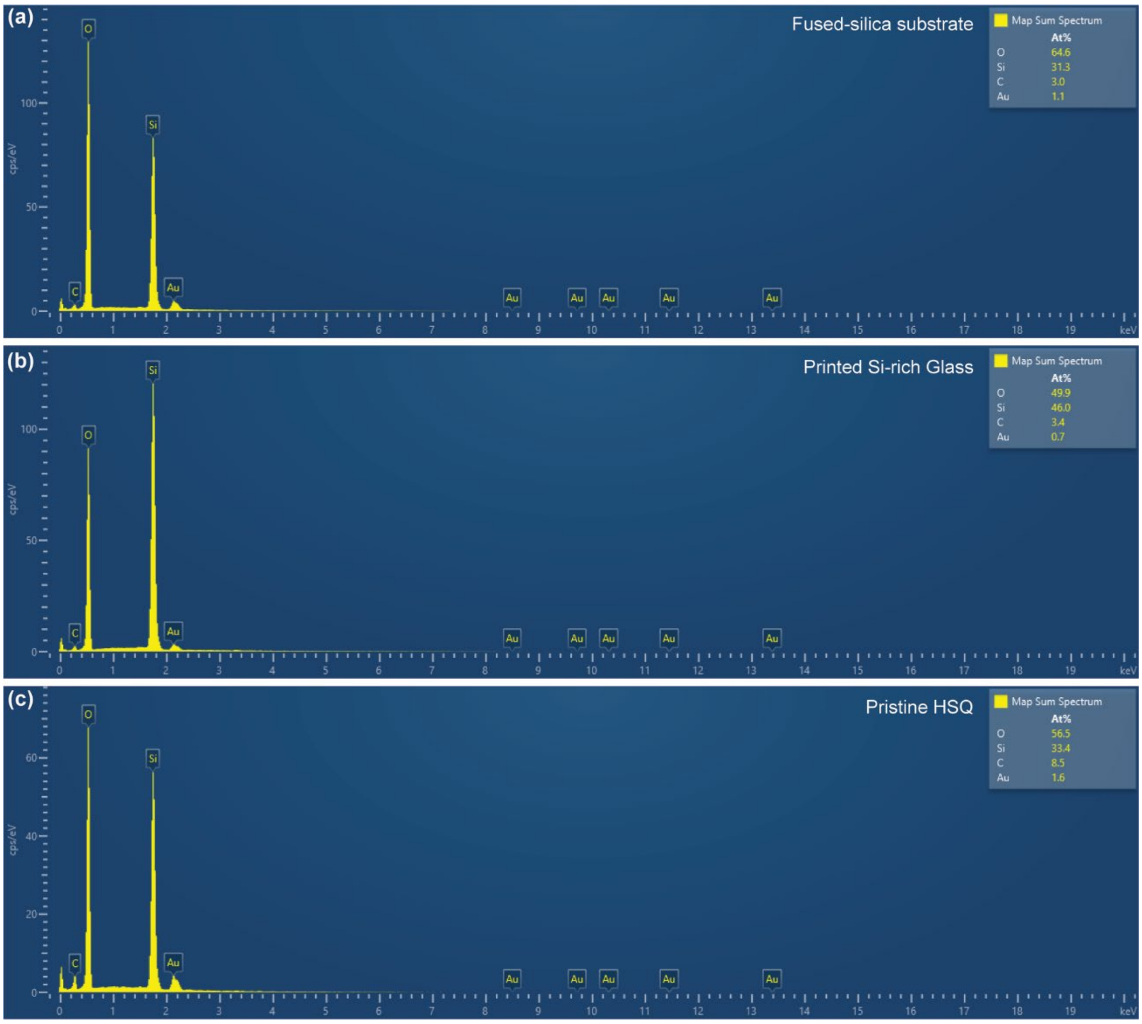

Supporting Figure 3. Representative EDS spectra of fused-silica substrate, 3D-printed Si-rich glass, and HSQ, respectively. The spectra were measured of the fused-silica substrate (**a**), 3D-printed Si-rich glass (**b**), and pristine HSQ (**c**) on the same sample. The gold peaks observed in the spectra originated from the gold coating on the sample to prevent charging effects during the measurements.

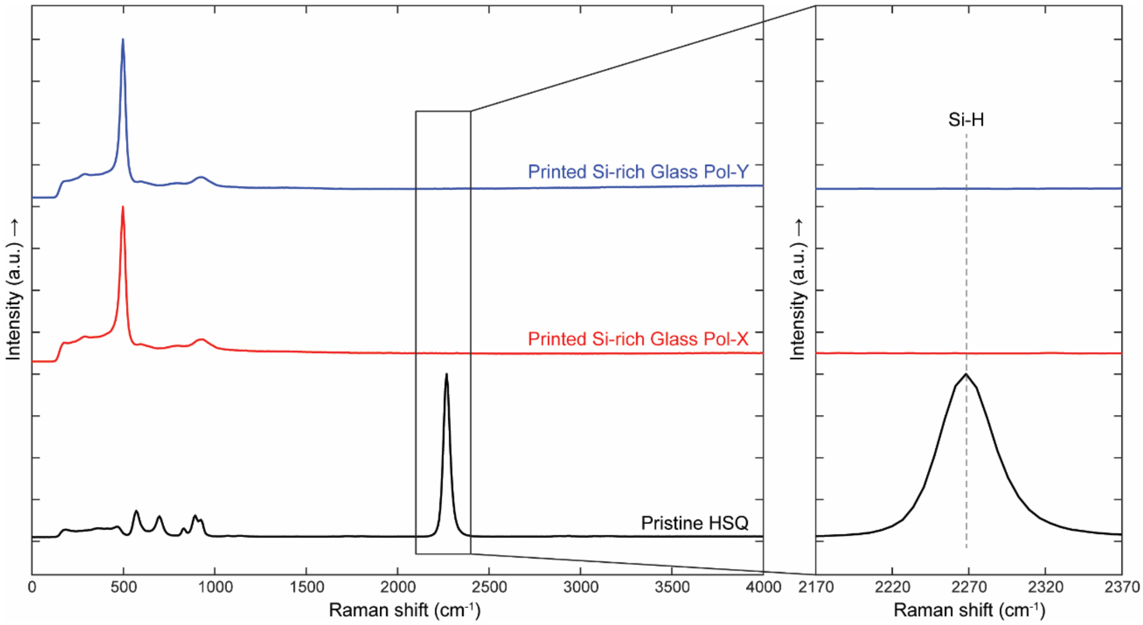

Supporting Figure 4. Extended Raman spectra of the 3D-printed Si-rich glass and the pristine HSQ. The spectra were measured of a pristine HSQ sample (black) and two Si-rich glass samples printed with the laser polarization in parallel (Pol-X, red) and perpendicular (Pol-Y, blue) to the laser writing direction, respectively. An enlarged view of the spectra around the Raman shifts of 2260 cm^{-1}, which is the spectral position of the signature Si-H peak of pristine HSQ, is provided to show that the Si-H Raman feature was not present in the 3D-printed Si-rich glass.

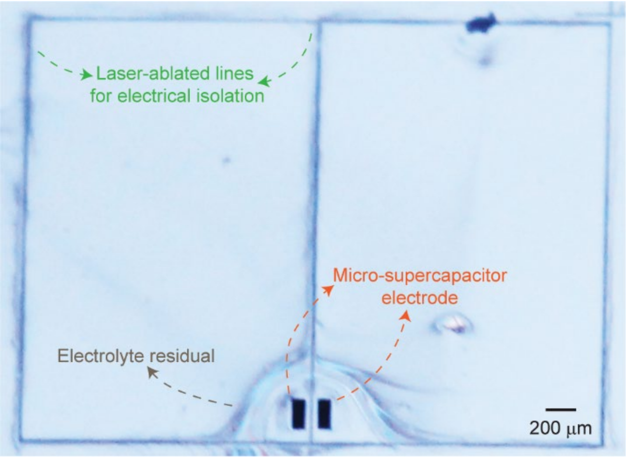

Supporting Figure 5. Photograph of a 3D-printed micro-supercapacitor. The photograph was taken using a camera (EOS 600D, Canon) after performing the cyclic voltammetry characterization of the micro-supercapacitor (MSC) and removal of the used gel electrolyte (i.e., poly(4-styrenesulfonic acid)/LiCl composite) by immersing the sample in deionized water. The electrodes appear dark in the photograph due to the scattering of light by the nanogratings in the 3D-printed structures. To electrically isolate the two electrodes of the MSC, femtosecond laser ablation was applied to selectively remove the deposited TiN layer which served as the current collector of the MSC. The areas between the laser-ablated lines and the MSC electrodes were designed to be relatively large to facilitate the placement of the probes during device characterization.

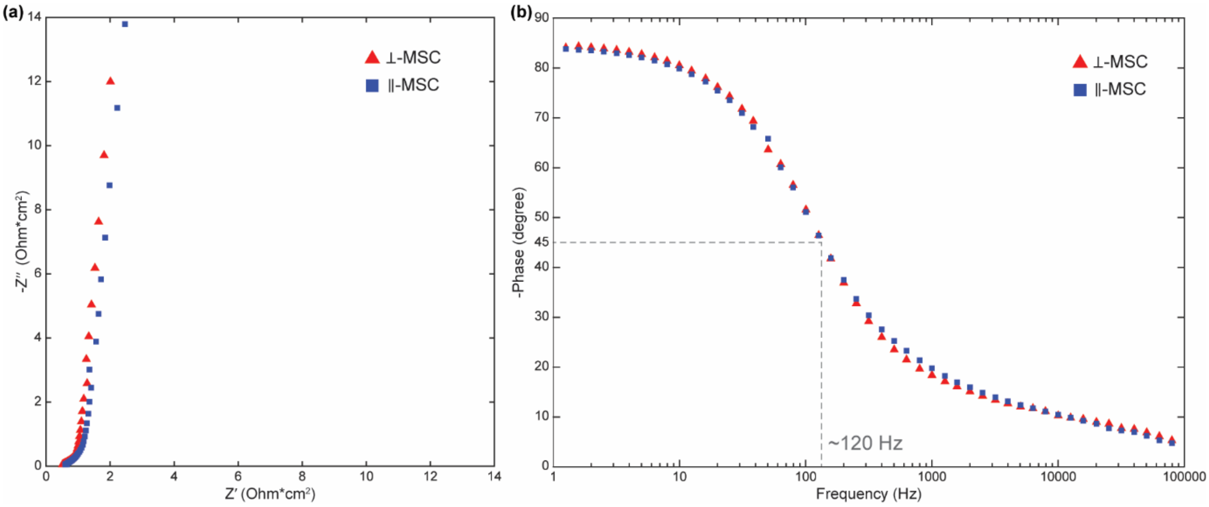

Supporting Figure 6. Characterization of the 3D-printed micro-supercapacitors (MSCs) using electrochemical impedance spectroscopy. (a) Nyquist plots measured from the 3D-printed MSCs shown in **Figure 4** in a frequency range of 10 Hz to 200 kHz. **(b)** Bode plots measured from the 3D-printed MSCs shown in **Figure 4**. The characteristic frequency of each MSC is marked, which is the point at which the resistive and capacitive impedances are equal, i.e., phase equals to -45°.

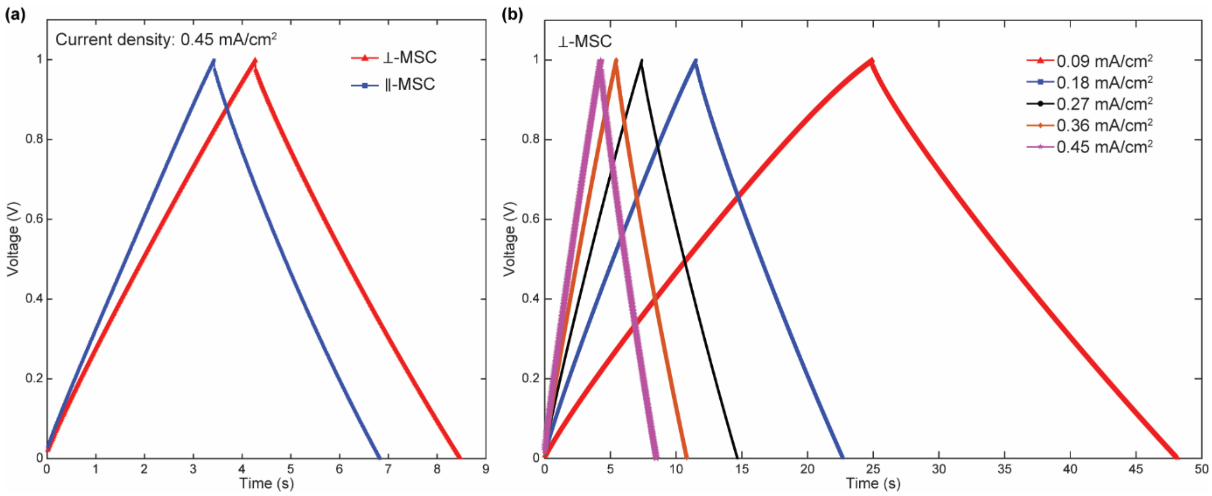

Supporting Figure 7. Galvanostatic charge–discharge (GCD) curves measured from the 3D-printed micro-supercapacitors (MSCs). (a) GCD curves measured from the 3D-printed MSCs shown in **Figure 4** at a current density of 0.45 mA/cm^2. **(b)** GCD curves measured from the ⊥-MSC shown in **Figure 4** at various current densities between 0.09 mA/cm^2 and 0.45 mA/cm^2.

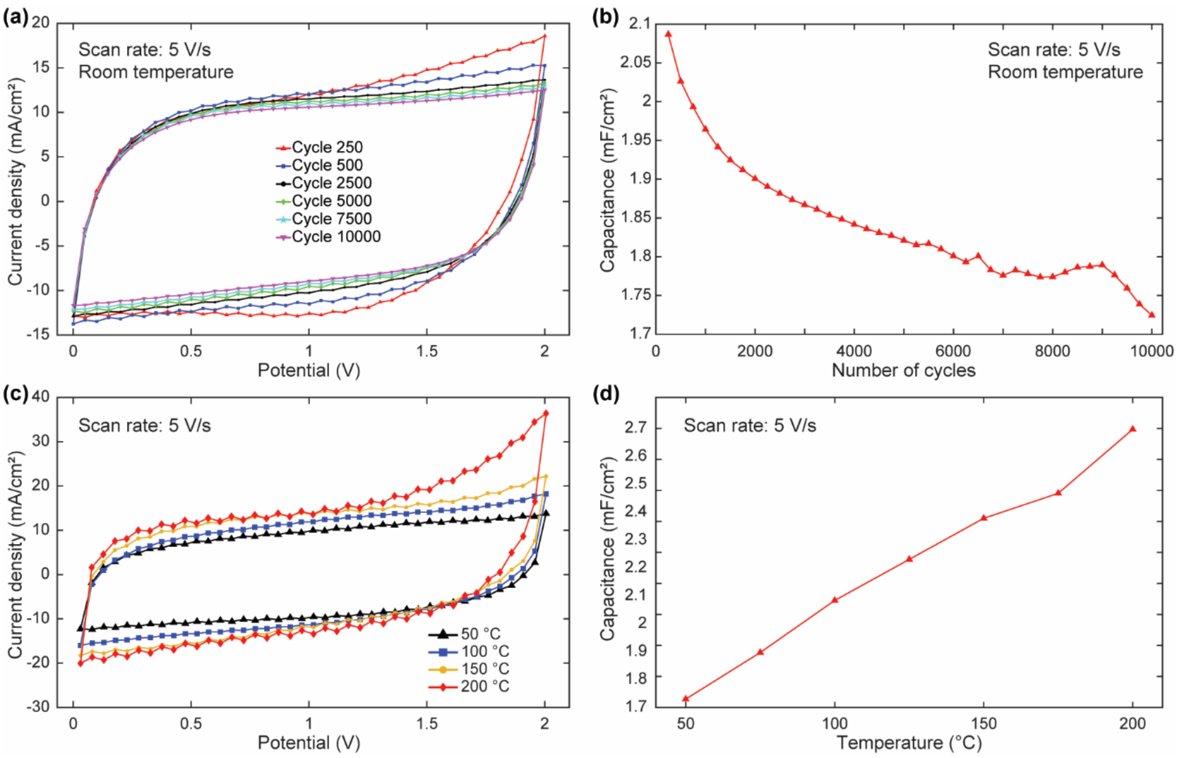

Supporting Figure 8. Long-cycling and high-temperature experiments of a 3D-printed Si-rich glass micro-supercapacitor with an ionic-liquid electrolyte. (a) Measured cyclic-voltammetry (CV) curves of the 3D-printed micro-supercapacitor after different charging-discharging cycles at room temperature. **(b)** Areal capacitances extracted from the corresponding measured CV curves of the 3D-printed micro-supercapacitor after different charging-discharging cycles at room temperature. **(c)** Measured CV curves of the 3D-printed micro-supercapacitor at different temperatures. **(d)** Areal capacitances extracted from the corresponding measured CV curves of the 3D-printed micro-supercapacitor at different temperatures.

Supporting Table 1. Comparison of high-scan-rate supercapacitors in literature.

Supercapacitor	Capacitance	Voltage window	Electrolyte	Reference
MXene (electrode only)	100 mF cm^{-2} @50 V s^{-1}	0-0.6 V	Liquid H_2SO_4	8
r-GO/TiO	0.35 mF cm^{-2} @50 V s^{-1}	0-0.8 V	PVA/H_2SO_4	9
Graphene	0.021 mF cm^{-2} @50 V s^{-1}	0-1 V	PVA/H_2SO_4	10
Graphene	0.23 mF cm^{-2} @10 V s^{-1}<br>0.15 mF cm^{-2} @100 V s^{-1}	0-1 V	PVA/H_2SO_4	11
MXene/PEDOT	0.62 mF cm^{-2} @50 V s^{-1}	0-0.8 V	c-PVA/H_2SO_4	12
Graphene/Au	0.11 mF cm^{-2} @50 V s^{-1}	0-0.8 V	PVA/H_2SO_4	13
Graphene	0.86 mF cm^{-2} @50 V s^{-1}	0-1 V	PVA/H_2SO_4	14
Graphene	0.082 mF cm^{-2} @10 V s^{-1}<br>0.032 mF cm^{-2} @100 V s^{-1}	0-1 V	PVA/H_2SO_4	15
2D rGO/Au<br>3D rGO/Au	1 mF cm^{-2} @50 V s^{-1}<br>3 mF cm^{-2} @50 V s^{-1}	0-1 V	PVA/H_2SO_4	16
N/B co-doped graphene	0.063 mF cm^{-2} @50 V s^{-1}	0-1 V	PVA/H_2SO_4	17
PANI/Graphene	0.34 mF cm^{-2} @50 V s^{-1}	0-0.8 V	Liquid H_2SO_4	18
Graphene	0.021 mF cm^{-2} @50 V s^{-1}	0-1 V	PVA/H_2SO_4	19
S doped Graphene	0.06 mF cm^{-2} @40 V s^{-1}	0-1 V	PVA/H_2SO_4	20
Graphene Nanoribbons	1.64 mF cm^{-2} @10 V s^{-1}<br>0.7 mF cm^{-2} @100 V s^{-1}	0-1 V	PVA/H_2SO_4	21
MXene	1.96 mF cm^{-2} @20 V s^{-1}<br>1.6 mF cm^{-2} @80 V s^{-1}	0-0.6 V	PVA/H_2SO_4	22
Carbon onions	0.9 mF cm^{-2} @100 V s^{-1}	0-3 V	Et4NBF4/anhydrous propylene carbonate electrolyte	23
**TiN/Si-rich glass nanoplates**	**1 mF cm^{-2} @50 V s^{-1}**<br>**2.09 mF cm^{-2} @5 V s^{-1}**	**0-1 V**<br>**0-2 V**	**PSSH/LiCl**<br>**BMIM-BF4**	**This work**

Reference

(1) Chan, J. W.; Huser, T. R.; Risbud, S. H.; Krol, D. M. Modification of the Fused Silica Glass Network Associated with Waveguide Fabrication Using Femtosecond Laser Pulses. *Appl. Phys. A: Mater. Sci. Process.* **2003**, *76* (3), 367–372.

(2) Volodin, V. A.; Koshelev, D. I. Quantitative Analysis of Hydrogen in Amorphous Silicon Using Raman Scattering Spectroscopy. *J. Raman Spectrosc.* **2013**, *44* (12), 1760–1764.

(3) Bell, R. J.; Bird, N. F.; Dean, P. The Vibrational Spectra of Vitreous Silica, Germania and Beryllium Fluoride. *J. Phys. C: Solid State Phys.* **1968**, *1* (2), 299–303.

(4) Salh, R. Defect Related Luminescence in Silicon Dioxide Network: A Review. In *Crystalline Silicon - Properties and Uses*; Basu, S., Ed.; InTech, 2011; pp 135–172. https://doi.org/10.5772/22607.

(5) Glinka, Y. D.; Lin, S. H.; Chen, Y. T. The Photoluminescence from Hydrogen-Related Species in Composites of SiO2 Nanoparticles. *Appl. Phys. Lett.* **1999**, *75* (6), 778–780.

(6) Nesheva, D.; Raptis, C.; Perakis, A.; Bineva, I.; Aneva, Z.; Levi, Z.; Alexandrova, S.; Hofmeister, H. Raman Scattering and Photoluminescence from Si Nanoparticles in Annealed SiO x Thin Films. *J. Appl. Phys.* **2002**, *92* (8), 4678–4683.

(7) Yu, Z.; Aceves, M. Photoluminescence in Off-Stoichiometric Silicon Oxide Compounds. *Superficies y vacío* **2004**, *17* (1), 1–6.

(8) Xia, Y.; Mathis, T. S.; Zhao, M. Q.; Anasori, B.; Dang, A.; Zhou, Z.; Cho, H.; Gogotsi, Y.; Yang, S. Thickness-Independent Capacitance of Vertically Aligned Liquid-Crystalline MXenes. *Nature* **2018**, *557* (7705), 409–412.

(9) Wang, S.; Wu, Z.-S.; Zheng, S.; Zhou, F.; Sun, C.; Cheng, H.-M.; Bao, X. Scalable Fabrication of Photochemically Reduced Graphene-Based Monolithic Micro-Supercapacitors with Superior Energy and Power Densities. *ACS Nano* **2017**, *11* (4), 4283–4291.

(10) Wu, Z.; Parvez, K.; Feng, X.; Müllen, K. Graphene-Based in-Plane Micro-Supercapacitors with High Power and Energy Densities. *Nat. Commun.* **2013**, *4* (1), 2487.

(11) Wu, Z.-S.; Liu, Z.; Parvez, K.; Feng, X.; Müllen, K. Ultrathin Printable Graphene Supercapacitors with AC Line-Filtering Performance. *Adv. Mater.* **2015**, *27* (24), 3669–3675.

(12) Gund, G. S.; Park, J. H.; Harpalsinh, R.; Kota, M.; Shin, J. H.; Kim, T.; Gogotsi, Y.; Park, H. S. MXene/Polymer Hybrid Materials for Flexible AC-Filtering Electrochemical Capacitors. *Joule* **2019**, *3* (1), 164–176.

(13) Kwon, S.; Lee, T.; Choi, H.-J.; Ahn, J.; Lim, H.; Kim, G.; Choi, K.-B.; Lee, J. Scalable Fabrication of Inkless, Transfer-Printed Graphene-Based Textile Microsupercapacitors with High Rate Capabilities. *J. Power Sources* **2021**, *481*, 228939.

(14) Zhang, L.; DeArmond, D.; Alvarez, N. T.; Malik, R.; Oslin, N.; McConnell, C.; Adusei,

P. K.; Hsieh, Y.; Shanov, V. Flexible Micro-Supercapacitor Based on Graphene with 3D Structure. *Small* **2017**, *13* (10), 1603114.

(15) Liu, Z.; Chen, Z.; Wang, C.; Wang, H. I.; Wuttke, M.; Wang, X.-Y.; Bonn, M.; Chi, L.; Narita, A.; Müllen, K. Bottom-Up, On-Surface-Synthesized Armchair Graphene Nanoribbons for Ultra-High-Power Micro-Supercapacitors. *J. Am. Chem. Soc.* **2020**, *142* (42), 17881–17886.

(16) Li, R.-Z.; Peng, R.; Kihm, K. D.; Bai, S.; Bridges, D.; Tumuluri, U.; Wu, Z.; Zhang, T.; Compagnini, G.; Feng, Z.; Hu, A. High-Rate in-Plane Micro-Supercapacitors Scribed onto Photo Paper Using in Situ Femtolaser-Reduced Graphene Oxide/Au Nanoparticle Microelectrodes. *Energy Environ. Sci.* **2016**, *9* (4), 1458–1467.

(17) Wu, Z.-S.; Parvez, K.; Winter, A.; Vieker, H.; Liu, X.; Han, S.; Turchanin, A.; Feng, X.; Müllen, K. Layer-by-Layer Assembled Heteroatom-Doped Graphene Films with Ultrahigh Volumetric Capacitance and Rate Capability for Micro-Supercapacitors. *Adv. Mater.* **2014**, *26* (26), 4552–4558.

(18) Liu, Z.; Liu, S.; Dong, R.; Yang, S.; Lu, H.; Narita, A.; Feng, X.; Müllen, K. High Power In-Plane Micro-Supercapacitors Based on Mesoporous Polyaniline Patterned Graphene. *Small* **2017**, *13* (14), 1603388.

(19) Ye, J.; Tan, H.; Wu, S.; Ni, K.; Pan, F.; Liu, J.; Tao, Z.; Qu, Y.; Ji, H.; Simon, P.; Zhu, Y. Direct Laser Writing of Graphene Made from Chemical Vapor Deposition for Flexible, Integratable Micro-Supercapacitors with Ultrahigh Power Output. *Adv. Mater.* **2018**, *30* (27), 1801384.

(20) Wu, Z.-S.; Tan, Y.-Z.; Zheng, S.; Wang, S.; Parvez, K.; Qin, J.; Shi, X.; Sun, C.; Bao, X.; Feng, X.; Müllen, K. Bottom-Up Fabrication of Sulfur-Doped Graphene Films Derived from Sulfur-Annulated Nanographene for Ultrahigh Volumetric Capacitance Micro-Supercapacitors. *J. Am. Chem. Soc.* **2017**, *139* (12), 4506–4512.

(21) Liu, Z.; Hu, Y.; Zheng, W.; Wang, C.; Baaziz, W.; Richard, F.; Ersen, O.; Bonn, M.; Wang, H. I.; Narita, A.; Ciesielski, A.; Müllen, K.; Samorì, P. Untying the Bundles of Solution-Synthesized Graphene Nanoribbons for Highly Capacitive Micro-Supercapacitors. *Adv. Funct. Mater.* **2022**, *32* (16).

(22) Wang, S.; Li, L.; Zheng, S.; Das, P.; Shi, X.; Ma, J.; Liu, Y.; Zhu, Y.; Lu, Y.; Wu, Z.-S.; Cheng, H.-M. Monolithic Integrated Micro-Supercapacitors with Ultra-High Systemic Volumetric Performance and Areal Output Voltage. *Natl. Sci. Rev.* **2023**, *10* (3).

(23) Pech, D.; Brunet, M.; Durou, H.; Huang, P.; Mochalin, V.; Gogotsi, Y.; Taberna, P.-L.; Simon, P. Ultrahigh-Power Micrometre-Sized Supercapacitors Based on Onion-like Carbon. *Nat. Nanotechnol.* **2010**, *5* (9), 651–654.
